# HDAC inhibitor PAC-320 induces G2/M cell cycle arrest and apoptosis in human prostate cancer

**DOI:** 10.18632/oncotarget.23070

**Published:** 2017-12-08

**Authors:** Zhixiong Dong, Yang Yang, Shuxia Liu, Jun Lu, Baiqu Huang, Yu Zhang

**Affiliations:** ^1^ Institute of Genetics and Cytology, The Key Laboratory of Molecular Epigenetic of Ministry of Education (MOE), Northeast Normal University, Changchun 130024, China; ^2^ Tianjin Key Laboratory of Animal and Plant Resistance, College of Life Sciences, Tianjin Normal University, Tianjin 300387, China; ^3^ The Key Laboratory of Polyoxometalates Science of Ministry of Education (MOE), College of Chemistry, Northeast Normal University, Changchun 130024, China; ^4^ Department of Biochemistry and Molecular Biology, Beijing Normal University, Beijing Key Laboratory, Beijing 100875, China

**Keywords:** PAC-320, HDAC inhibitor, prostate tumor, apoptosis

## Abstract

HDAC inhibitors (HDACis) have been demonstrated with profound antiproliferative activities in various tumor types. Previously, we screened several polyoxometalate HDACis based on our *p21* luciferase promoter system and demonstrated that such HDACis have antitumor activity. Here, we further investigate the antitumor mechanism of PAC-320, a compound among the polyoxometalates, in human prostate cancer. We demonstrate that PAC-320 is a broad-spectrum HDACi and could inhibit growth of prostate cancer cells *in vitro* and *in vivo*. Furthermore, we find that PAC-320 induces cell cycle arrest at G2/M phase and apoptosis. Mechanically, PAC-320 induced cell cycle arrest is associated with an increase of p21 and decrease of cyclin A and cyclin B1, while PAC-320 induced apoptosis is mediated through mitochondria apoptotic pathway and is closely associated with increase of BH3-only proteins Noxa and Hrk. Meanwhile, we demonstrate that p38 MAPK pathway is involved in PAC-320 induced antiproliferative activities in prostate cancer. Taken together, our data indicates that PAC-320 has potent prostate cancer inhibitory activity *in vitro* and *in vivo*, which is mediated by G2/M cell cycle arrest and apoptosis.

## INTRODUCTION

Prostate cancer is one of the most prevalent urological malignancies worldwide and a leading cause of cancer-related morbidity and mortality in men [1–3]. This cancer is highly dependent on the androgen receptor (AR) signaling pathway. Therefore, Androgen-deprivation therapy has been the mainstay treatment for advanced prostate cancer and induces remission in 80–90% of men with advanced disease, resulting in a median disease progression-free survival of 12–33 months [4]. Unfortunately, most of patients subsequently acquire resistance to those deprivation therapies, and result in castration-resistant prostate cancers (CRPC), which have a high mortality rate [5]. Therefore, there is a great need to develop better therapies for prostate cancer. Prostate cancer is a heterogeneous disease, the etiology of which appears to be related to a complex range of risk factors, including lifestyle patterns, genetic factors and epigenetic modifications [6]. These abnormal epigenetic modifications associated prostate cancer include DNA methylation, histone methylation and histone acetylation, etc. For example, HDAC1 (Histone deacetylase 1), HDAC2 and HDAC3 are strongly expressed in prostate cancer [7, 8].

It has been widely recognized in recent years that HDACs are promising targets for therapeutic interventions intended to reverse aberrant epigenetic states associated with cancer [9]. Consequently, there has been considerable effort to develop HDACis. HDACis can reverse the activity of HDACs, since recruitment of HDACs predominantly leads to transcriptional repression, HDACis can reverse repression in transformed cells and lead to re-expression of genes inducing cell cycle arrest, differentiation and/or cell death, but have lower toxicity to normal cells [10, 11]. Several small synthetic and natural HDACis have advanced into clinical trials [11, 12]. Such compounds show single-agent safety, pharmacodynamic biomarker induction, and evidence of antitumor activity in a variety of hematologic and solid cancers. Moreover, Vorinostat (SAHA) and romidepsin (FK228) have been approved by FDA and EMEA in treatment of uncontrolled cutaneous T cell lymphoma, thus providing clinical validation of this therapeutic strategy [13–16]. However, most of the compounds in clinical development seem to have limitations, including low potency, undesirable safety profiles that include cardiovascular safety issues, and potential for drug-drug interactions via cytochrome P450 inhibition [17]. Hence, there remains a significant clinical opportunity for efficacious HDACis which are safe and well tolerated.

Previously, we screened 13 Polyoxometalate (POM) compounds using a *p21* promoter luciferase report gene system and demonstrated that those compounds had HDACi activity and inhibited proliferation of various cancer cells [18]. However, the antitumor mechanisms of these new POM based HDACis are unclear. Here, we further investigated the antitumor activity and mechanisms of action of PAC-320, the most effective POM based HDACis, in prostate cancer cells.

## RESULTS

### PAC-320 inhibits class I and class II HDACs activity and induces histone hyperacetylation in prostate cancer cells.

**Figure 1:**
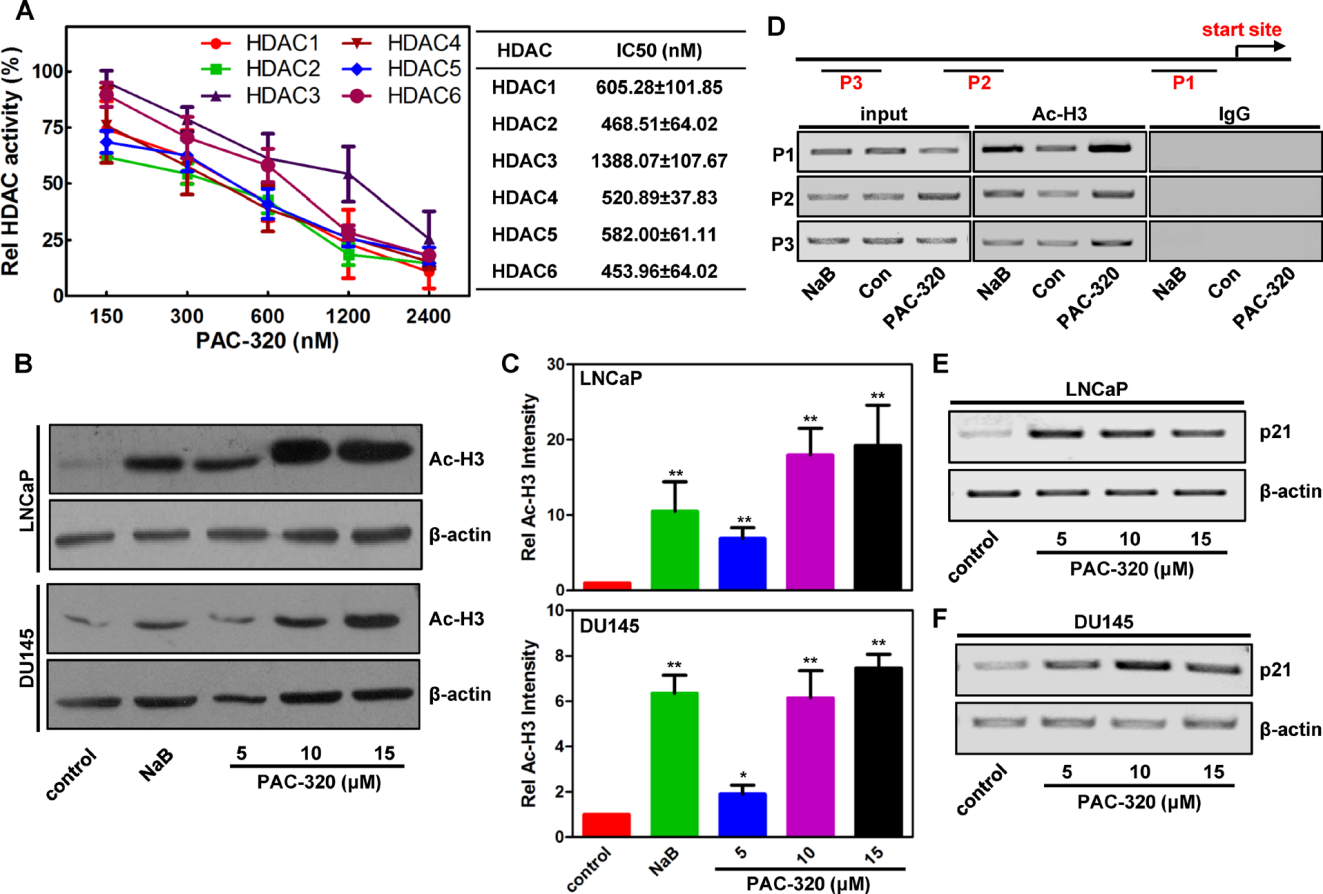
PAC-320 is broad-spectrum HDACi (**A**) *In vitro* HDAC inhibition assay. HDAC activity was analyzed at different PAC-320 concentrations by measuring HDAC substrate fluorescence. Diluted HDAC inhibitor and substrate was added. Reactions were performed as described in “Materials and Methods”. Fluorescence was analyzed using a luminescence spectrometer. Results are shown as means based on experiments performed in triplicate; bars, ±SD. (**B**) *In vivo* HDAC inhibition assay. Immunoblotting analyze the effect of PAC-320 on acetylation of histone H3 in LNCaP or DU145 cells. NaB as a positive control. Amounts of immunoblotted proteins were quantified by normalized to β-actin. (**C**) Histograms represented the level of acetylated H3 after HDACi treatment relative to control. Results are representative of three independent experiments. bars indicate SD. *P < 0.05; **P < 0.01. (**D**) PAC-320 regulates H3 acetylation of *p21* promoter. DU145 cells were treated with 10 μM PAC-320 for 24 h and harvested for ChIP assays. Samples were immunoprecipitated with α-acetyl H3, and the precipitated DNA fragments were amplified by PCR using specific primers as indicated in the diagram of *p21* promoter. (**E**–**F**) PAC-320 upregulates p21 expression at transcriptional level. LNCaP (E) or DU145 (F) cells were treated with PAC-320 at indicated concentrations for 48 h. The mRNA was extracted and amplified by RT-PCR using specific primers.

In previous study, we demonstrated that PAC-320 has HDAC inhibitor activity by HDAC activity assay *in vitro* [18]. Here, we further analyzed the target specificities of PAC-320 in detail. We performed an *in vitro* HDAC inhibition assay on each HDAC isotype. As can be seen in Figure 1A, PAC-320 significantly inhibited the enzyme activity of HDAC1, 2, 4, 5 and 6, but to a less extent, HDAC 3. It was shown that PAC-320 has an IC_50_ range from 0.45–1.39 μM to each HDAC isotype. These results suggest that PAC-320 is a broad-spectrum HDACi that inhibit both class I and class II HDAC activity at micromole concentration.

To further confirm the ability of PAC-320 in inhibiting HDAC activity in human prostate cancer cells, we performed immunoblot analysis to determine its effects on the level of acetylated H3 (Ac-H3). LNCaP, DU145 or PC3 cells were treated with various doses of PAC-320 or 1mM sodium butyrate (NaB, a known HDACi), and histones extracted from nuclei were then subjected to immunoblot analysis. As shown in Figure 1B, 1C and Supplementary Figure 1A, control cells showed low basal levels of acetylated H3. However, similar to NaB, treatment with PAC-320 induced hyperacetylation of H3 in a dose-dependent manner. The cellular effect of PAC-320 on nuclear histone acetylation correlated well with the cell-free *in vitro* effects of PAC-320 on HDAC activity.

p21 is generally considered as a target of HDACis. Meanwhile, PAC-320 was screened using a cell-based screening system targeting *p21* gene promoter. Therefore, we further examined the acetylation status of *p21* promoter following PAC-320 treatment. DU145 cells were treated with or without PAC-320, and cells were collected for ChIP assay using α-acetyl H3 (Figure 1D). The ChIP results demonstrated that, compared with control, treatment with PAC-320 significantly increased the level of histone H3 acetylation at *p21* promoter in DU145 cells. Consistently, treatment with PAC-320 also induced an increase of p21 mRNA in LNCaP or DU145 cells in a dose-dependent manner (Figure 1E and 1F). These results demonstrate that PAC-320 could inhibit HDACs activity and enhances the acetylation of histones around the promoter region of *p21*, thereby upregulates p21 mRNA in prostate cancer cells.

### PAC-320 exhibits antitumor activities against human prostate cancer cells *in vitro* and *in vivo*

**Figure 2:**
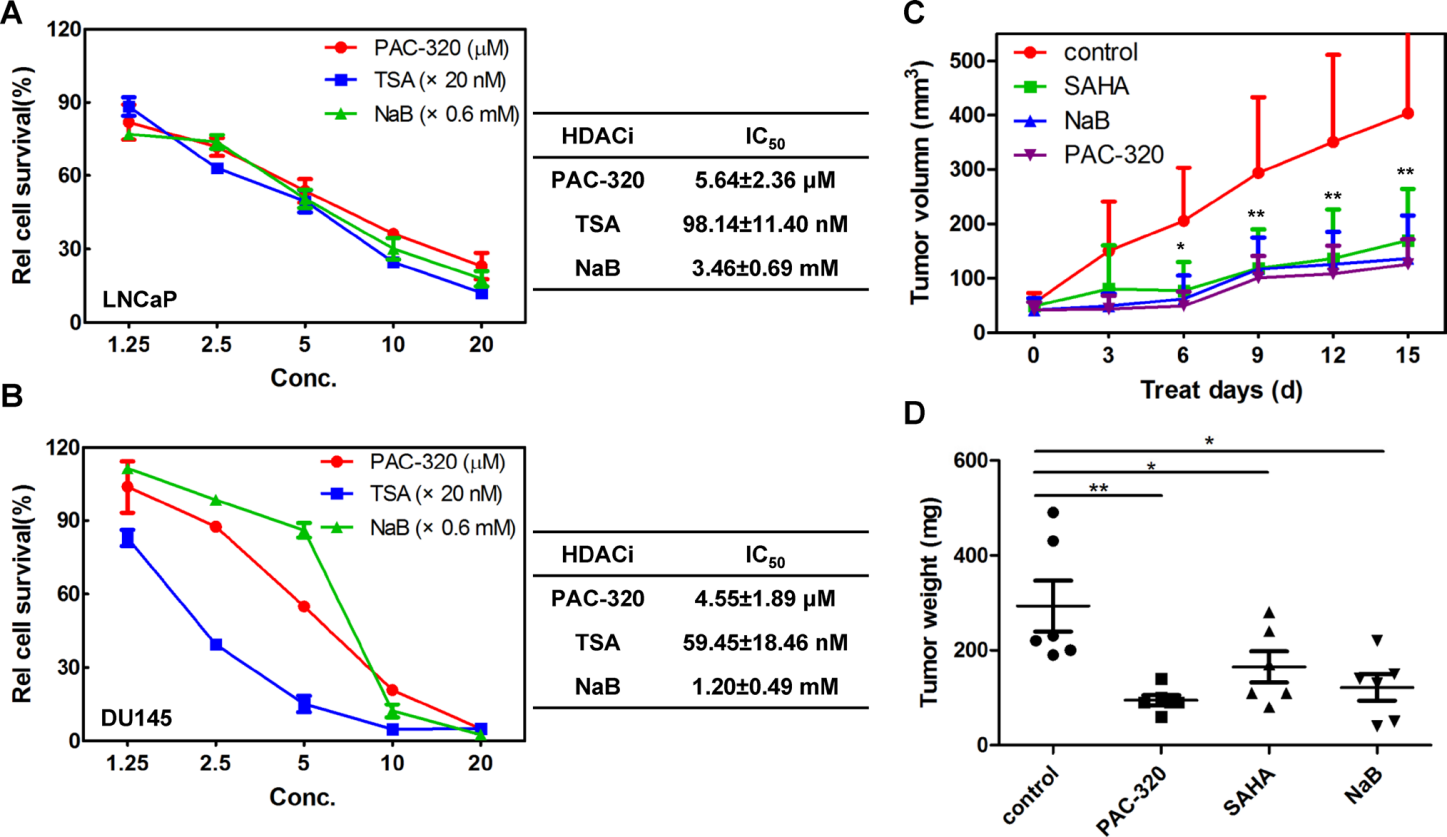
Antiproliferative effect of PAC-320 on prostate cancer cells (**A**–**B**) PAC-320 inhibits the proliferation of LNCaP (A) and DU145 (B) cells in a dose-dependent manner *in vitro*. LNCaP or DU145 cells were treated with indicated drugs for 72 h, and the effects of PAC-320 on proliferation of human prostate cancer cell lines were determined using the MTT assay and IC_50_ values were calculated using SPSS 21.0. TSA and NaB were used as positive controls. Each value represents the mean of three experiments; bars, ± SD. (**C**) *In vivo* antitumor activity of PAC-320 in prostate cancer DU145 xenograft models. Drugs were administered intraperitoneally into mice bearing tumors daily for 16 days. Tumor growth curves of DU145 cells in BALB/c nude mice were determined as described in “Materials and Methods” (*n* = 6, error bars indicate SD). (**D**) All mice were sacrificed on day 17 and the tumors were dissected and weighed.*P < 0.05; **P < 0.01.

HDACis have been shown to have potent antitumor activity in human cancer cells [12, 19–21]. Thus, we examined the effects of PAC-320 on the growth of human prostate cancer cells. LNCaP (AR positive), DU145 or PC3 (both are AR negative) cells were treated with various doses of PAC-320, trichostatin A (TSA) or NaB for 72 h, and cell viability was determined by MTT assay. As shown in Figure 2A–2B and Supplementary Figure 1B, the growth of LNCaP or DU145 cells was significantly inhibited in the presence of PAC-320 in a dose-dependent manner. In contrast, the growth-inhibitory effect of PAC-320 to PC3 cells was weaker than LNCaP or DU145 cells. The cytotoxicity of PAC-320 was also tested in comparison with the positive controls TSA and NaB, and the results revealed that PAC-320 has IC_50_ values at micromolar concentration in LNCaP or DU145 cells (Figure 2A and 2B). These results indicate that PAC-320 has potent antiproliferative activity against human prostate cancer cells *in vitro*.

To further evaluate the antitumor effect of PAC-320 against prostate cancer *in vivo*, we performed an animal study using a mouse model. We used DU145 model to assay *in vivo* antitumor activity of PAC-320. Nude mice bearing tumor xenografts were injected with PAC-320 (50 mg/kg), NaB (1 g/kg) or SAHA (40 mg/kg) daily for 16 days. The treatment did not appear to have a noticeable effect on body weight in mice. On average, PAC-320 treatment inhibited prostate tumors growth by 69.2% compared with that of control mice treated with only the vehicle (DMSO), while SAHA or NaB treatment was inhibited by 55.5% or 64.2% (Figure 2C). On day 17, mice were sacrificed and the tumor weights were measured. The weight of tumors was also significantly reduced in mice after treatment with PAC-320, SAHA or NaB (Figure 2D). Thus, PAC-320 displays significant antitumor activity in human prostate cancer xenograft model *in vivo*.

### PAC-320 induces cell cycle arrest at G2/M phase in human prostate cancer cells

**Figure 3:**
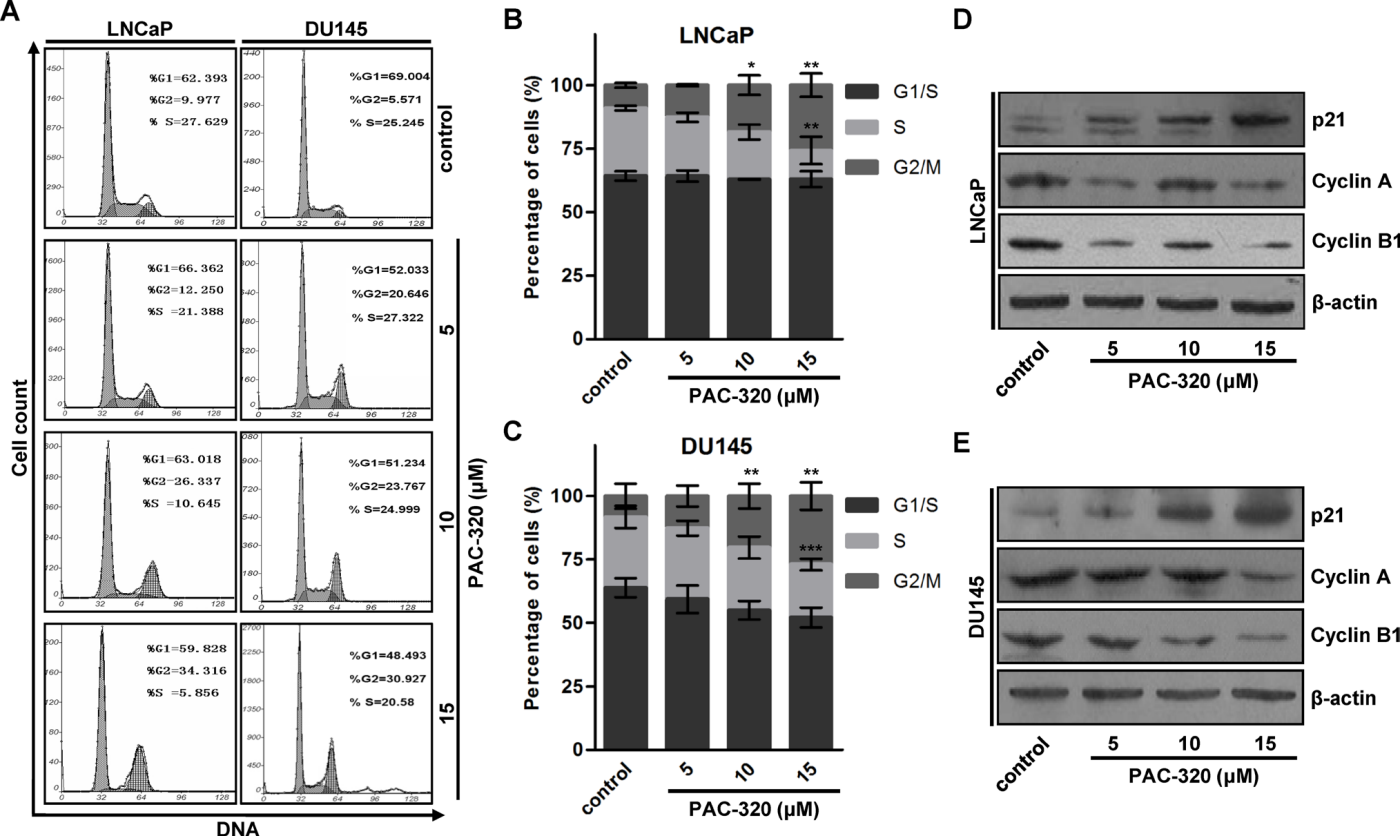
Antiproliferative effect of PAC-320 on prostate cancer cells involves cell cycle arrest at G2/M phase (**A**) Progression of cell cycle in PAC-320 treated cells. LNCaP or DU145 cells were treated with vehicle or indicated dose of PAC-320 for 48 h. After the indicated treatment times, the cells cycle profile was analyzed by FACS analysis. (**B**–**C**) The percentages of G1, S and G2/M phase cells were calculated from the DNA content histograms. The data represent the means from three independent experiments. bars, ± SD. *P < 0.05; **P < 0.01; ***P < 0.001. (**D**–**E**) PAC-320 regulates the expression of cell cycle related proteins. LNCaP (D) or DU145 (E) cells were treated with vehicle or various doses of PAC-320 for 48 h. Whole cell lysates were immunoblotted with indicated antibodies.

It was reported that the growth-inhibitory effects of HDACis are mediated by G1 and/or G2/M arrest and/or apoptosis [22–25]. Initially we analyzed cell cycle profiles after treating LNCaP or DU145 cells with various doses of PAC-320. Fluorescent-Activated Cell Sorting (FACS) analysis showed that, when compared with control, PAC-320 treatment caused an accumulation of cells in G2/M phase in both LNCaP and DU145 cells (Figure 3A–3C). These results indicate that PAC-320 could inhibit prostate cancer cells growth via inducing cell cycle arrest at G2/M phase.

To identify the molecules involved in PAC-320 induced cell cycle arrest, we examined a panel of cell cycle regulatory proteins by immunoblotting. We found that, consistent with previous results of p21 mRNA (Figure 1E and 1F), p21 protein was induced by PAC-320 treatment in a dose-dependent manner (Figure 3D and 3E). Cyclin A and cyclin B1, which are known to regulate the G2/M transition, were dramatically decreased after PAC-320 treatment in LNCaP or DU145 cells (Figure 3D and 3E). In addition, response to PAC-320 treatment, the expression of tumor suppressor p53 was decreased in LNCaP or DU145 cells (Supplementary Figure 2). These results suggest that PAC-320 induces G2/M phase arrest via upregulation of p21 and reduction of cyclin A and cyclin B1 in human prostate cancer cells.

### PAC-320 induces apoptosis in human prostate cancer cells

**Figure 4:**
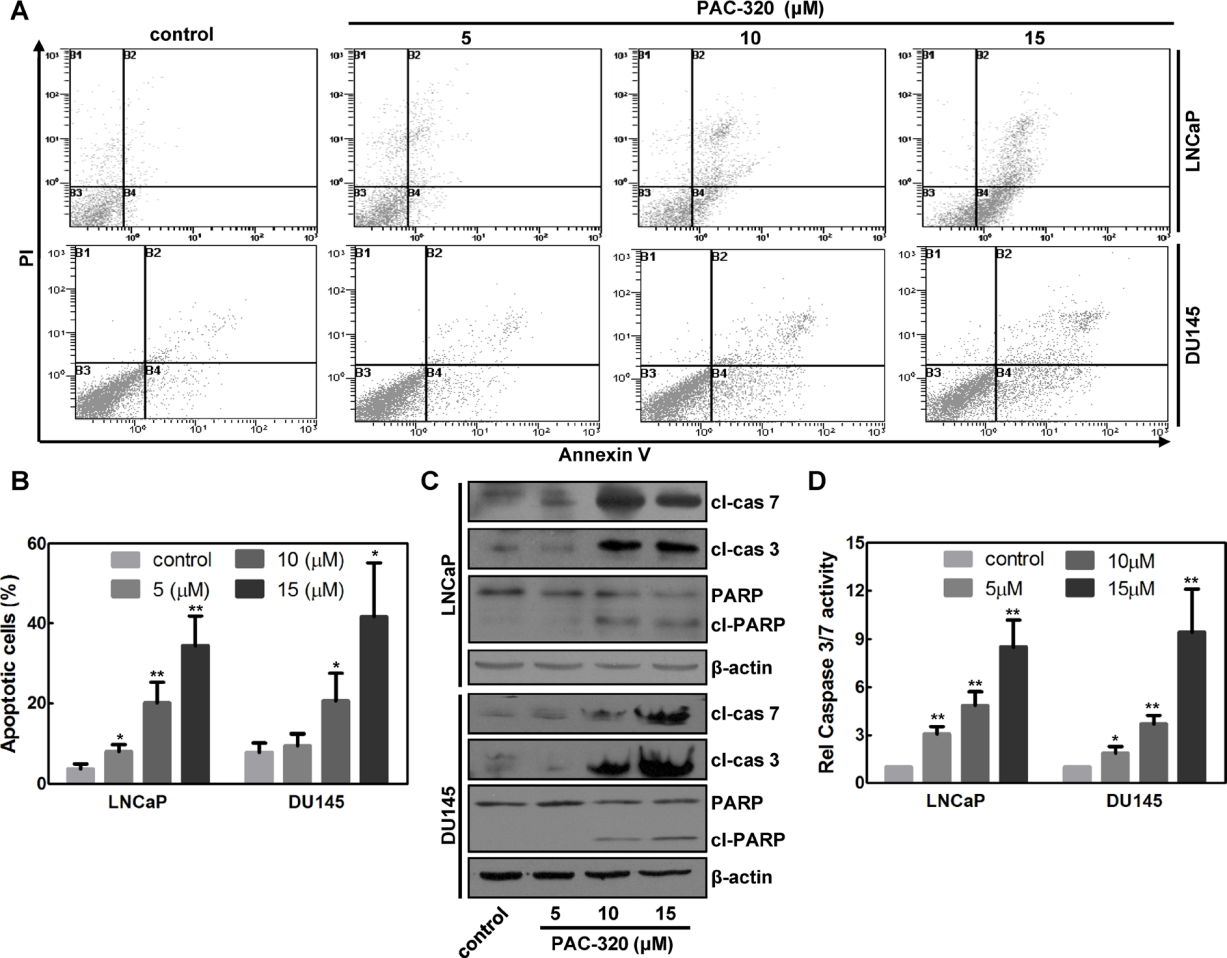
Antiproliferative effect of PAC-320 on prostate cancer cells involves apoptotic cell death (**A**) PAC-320 induces apoptosis in various human cancer cells. LNCaP or DU145 cells were treated with indicated concentrations of PAC-320 for 48 h. Cells were stained with Annexin V and PI and analyzed by FACS analysis. Representative flow cytometric dot plots showing an increased rate of apoptosis after PAC-320 treatment. (**B**) The data from three independent experiments in (A) are expressed as mean ± SD in the histogram. (**C**) PAC-320 induces caspase 3 and 7 activation and PARP cleavage. LNCaP or DU145 cells were treated with indicated concentrations of PAC-320 for 48 h. Whole cell lysates were immunoblotted with indicated antibodies. (**D**) PAC-320 induced caspase3/7 activities in LNCaP or DU145 cells. Cells incubated with indicated concentrations of PAC-320 for 48 h before caspase 3/7 activities were detected. The data from three independent experiments are expressed as mean ± SD in the histogram. *P < 0.05, **P < 0.01.

PAC-320 treatment also induced cell death. Then we want to examine whether PAC-320 induces apoptosis in prostate cancer cells. After 48 h of treatment with various doses of PAC-320, LNCaP or DU145 cells were co-stained with Annexin V and PI, and then analyzed by using FACS. As shown in Figure 4A and 4B, PAC-320 treatment significantly increased Annexin V positive cells in a dose-dependent manner, suggested that PAC-320 induced apoptosis in prostate cancer cells. We also confirmed the apoptotic cell death by examining caspase activation. Treatment with PAC-320 resulted in an increase in cleaved caspase 3 and 7 proteins with a concurrent increase in upregulation caspase 3/7 activity in LNCaP or DU145 cells (Figure 4C and 4D). Active caspase 3/7 cleaves a full-length poly (ADP-ribose)-polymerase (PARP; 116 kDa) into two fragments: (1) 89 kDa and (2) 24 kDa. Therefore, we further verified PAC-320 induced caspase 3/7 activation by checking the fragments of PARP. As shown in Figure 4C, an increase in the PARP 89 kDa cleavage product with the corresponding degradation of PARP 116 kDa protein was also observed in the cells after PAC-320 treatment. These results indicate that PAC-320 could inhibit prostate cancer cells growth via inducing cell apoptosis.

**Figure 5:**
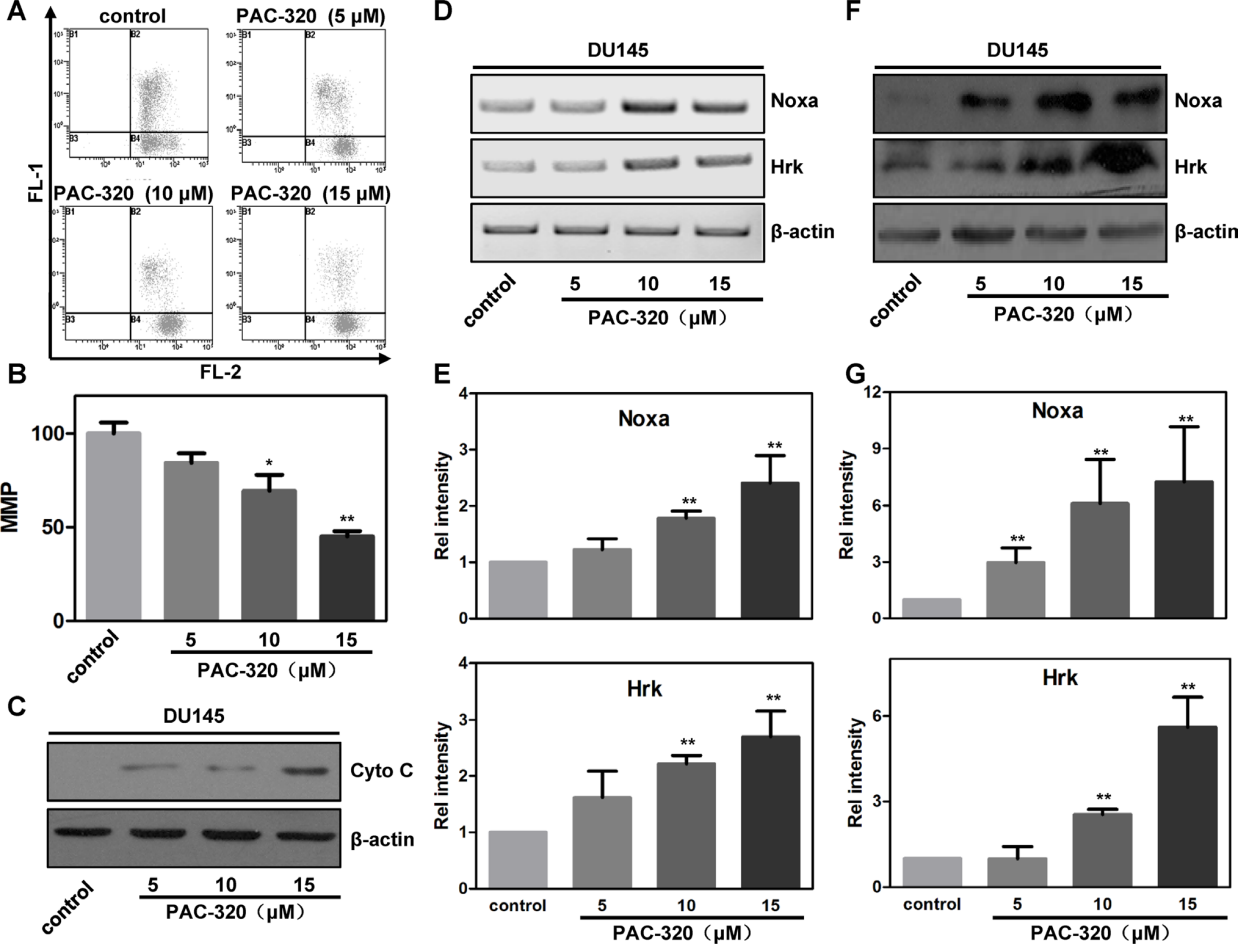
PAC-320 induces mitochondria mediated apoptosis in prostate cancer DU145 cells (**A**) PAC-320 induces decrease of mitochondrial membrane potential (MMP). DU145 cells were treated with indicated concentrations of PAC-320 for 48 h, and loss of MMP was determined by FACS as described in “Materials and Methods”. (**B**) The data from three independent experiments in (A) are expressed as mean ± SD in the histogram. (**C**) PAC-320 induces cytochrome c release to cytosol. Cells were treated as described in (A). Fractions of cytosol were isolated to examine the distribution of cytochrome c. β-actin was used as the marker for cytosol. (**D**–**G**) PAC-320 induces increases of BH3-only protein Noxa and Hrk. DU145 cells were treated with indicated concentrations of PAC-320 for 48 h. The expression of Noxa and Hrk were analyzed using RT-PCR (C) or Immunoblot (E). Histograms represented the level of mRNA (D) and protein (F) after PAC-320 treatment relative to control. The data represent the means from three independent experiments. bars indicated SD. *P < 0.05, **P < 0.01.

There are two distinct signaling pathways could lead to apoptotic cell death: (1) the extrinsic, or death receptor pathway, and (2) the intrinsic, or mitochondria mediated pathway [26, 27]. We could not observe any evidence of death receptor pathway activation after PAC-320 treatment (data not shown), which indicated that mitochondria pathway involved in PAC-320 induced apoptosis in prostate cancer cells. Therefore, we examined an important marker for mitochondria activity, the mitochondria membrane potential (MMP), in PAC-320 treated prostate cancer cells using JC-1 staining and FACS analysis. As shown in Figure 5A and 5B, the MMP of DU145 cells were significantly decreased after treatment with various doses of PAC-320. Loss of MMP initiates release of cytochrome c into the cytosol, which subsequently activates a caspase cascade that results in apoptosis. Thus, we separated mitochondrial and cytosolic fractions, and then examined the release of cytochrome c via immunoblot analysis. As shown in Figure 5C, treating the DU145 cells with PAC-320 resulted in a corresponding increase in cytochrome c in the cytosol. These results suggest that PAC-320 induces cell apoptosis through mitochondria mediated pathway.

Response to various exogenous and endogenous stresses, BH3-only family proteins play an important role in mitochondria mediated cell apoptosis [28, 29]. To test whether BH3-only family protein(s) participate in PAC-320 induced apoptosis in prostate cancer cells, we examined the expression of several BH3-only proteins using RT-PCR. Among them, the mRNA levels of Noxa and Hrk were significantly increased after treatment with various doses of PAC-320 in DU145 cells (Figure 5D–5E and Supplementary Figure 3A). Protein levels of Noxa and Hrk were also increased after PAC-320 treatment (Figure 5F–5G). Consistently, treatment with PAC-320 induced increase of Noxa and Hrk mRNA and protein level in LNCaP cells (Supplementary Figure 3B and 3C). Taken together, these results demonstrate that PAC-320 induces BH3-only proteins Noxa and Hrk expression which activate mitochondria mediated apoptosis in prostate cancer cells.

### p38 MAPK pathway is involved in PAC-320 induced proliferative inhibitory activity in human prostate cancer cells

**Figure 6:**
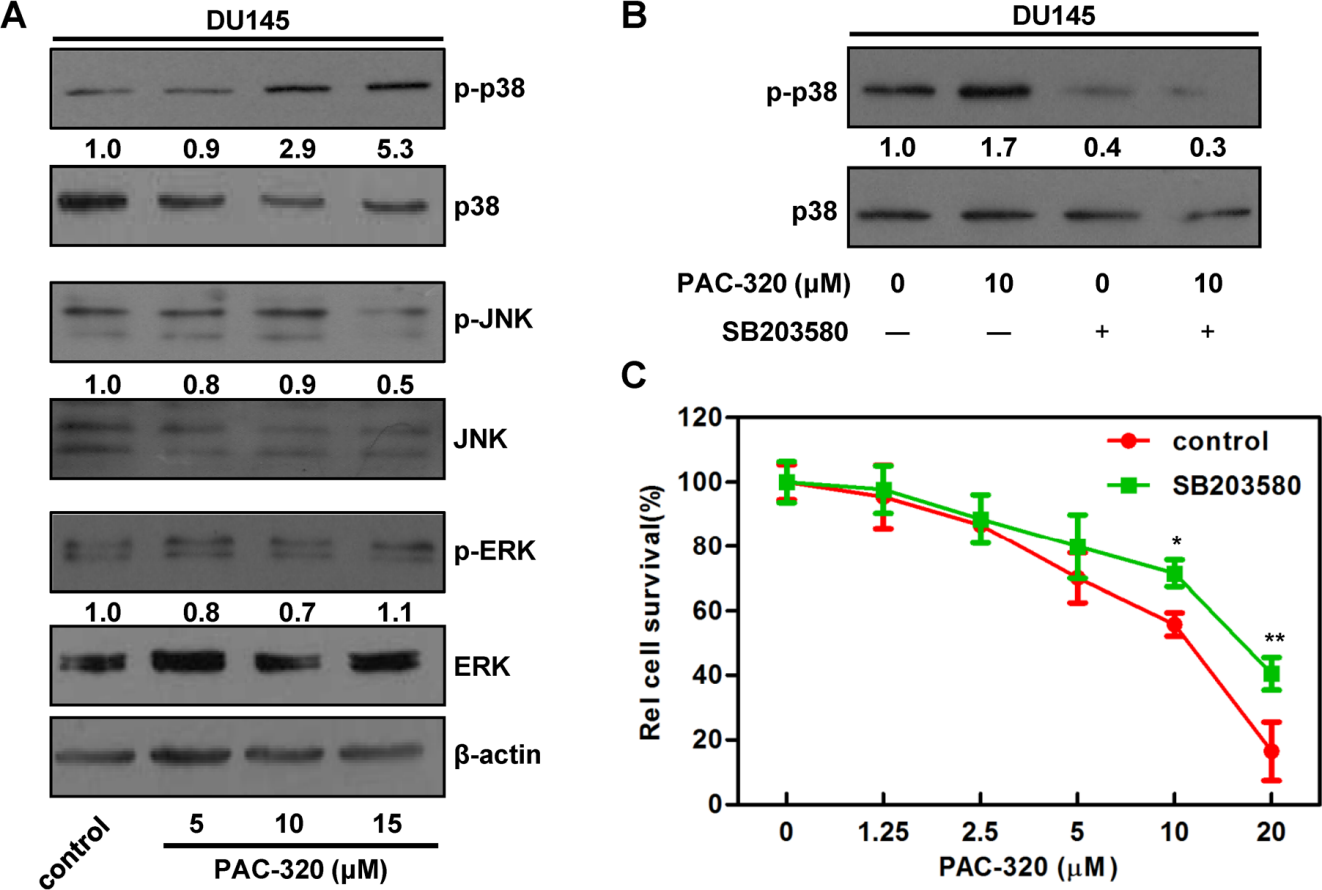
p38 MAPK signaling is involved in PAC-320 induced cell growth inhibition in DU145 cells (**A**) The effects of PAC-320 on MAPK phosphorylation. DU145 cells were treated with indicated concentrations of PAC-320 for 48 h, and whole cell lysates were immunoblotted with indicated antibodies. Numbers below the panels of the immunoblot indicated the protein levels relative to β-actin. (**B**) and (**C**) The effect of p38 MAPK inhibitor SB203580 on PAC-320 treatment effects. DU145 cells were treated with PAC-320 and/or 10 μM SB203580 for 48 h. The p38 phosphorylation or cell viability was determined by immunoblot (B) or MTT (C). Each value represents the mean of three experiments; bars, ± SD. *P < 0.05; **P < 0.01.

Several studies reported that MAPK pathway play an important role in HDACis induced proliferative inhibition [30–34]. To investigate if the pathway is involved in PAC-320 induced antitumor activity in prostate cancer cells, we detected the activation of MAP kinases, including extracellular signal-regulated kinase (ERK), c-Jun NH2-terminal kinase (JNK) and p38, after PAC-320 treatment (Figure 6A and Supplementary Figure 4). The protein levels of all 3 MAP kinases remained unchanged. However, the p38 phosphorylation was markedly increased after PAC-320 treatment in DU145 or LNCaP cells, but JNK and ERK phosphorylation remained the same as control (Figure 6A and Supplementary Figure 4). These data suggest that activation of p38 MAPK may be involved in PAC-320 induced antitumor activity in prostate cancer.

To explore the role of p38 MAPK pathway in PAC-320 induced cell proliferative inhibition, we pretreated DU145 cells with SB203580, an inhibitor of p38 MAPK pathway. Firstly, the cytotoxicity of SB203580 was tested. Results indicated that SB203580 alone did not cause significant growth inhibition in DU145 cells (data not shown). We next determined the effect of SB203580 on PAC-320 induced p38 activation and cell growth inhibition. Immunoblotting result indicated that SB203580 treatment decreased p38 phosphorylation in both PAC-320 treated and untreated cells (Figure 6B). Meanwhile, as indicated by MTT result, SB203580 treatment partially restored DU145 cell viabilities, compared with PAC-320 treatment alone (Figure 6C). These results demonstrate that p38 MAPK pathway partially mediates PAC-320 induced cell growth inhibition in prostate cancer cells.

## DISCUSSION

In this study, we investigate the antitumor activity and mechanism of action of PAC-320 against human prostate cancer cells *in vitro* and *in vivo*. We show that PAC-320 is a broad-spectrum HDACi and could effective inhibit proliferation of LNCaP or DU145 cells, but to a less extent, to PC3 cells. We further demonstrate that antiproliferative effect of PAC-320 on LNCaP or DU145 mainly due to G2/M cell cycle arrest and apoptosis.

Among the three cell lines we used, LNCaP or DU145 is more sensitive than PC3 to PAC-320 induced cell growth inhibition. It has been reported that antiapoptotic proteins Bcl-2 and Bcl-xl levels play an important role in PC3 resistance because blocking Bcl-2 family proteins increases the cells chemosensitivity [35]. DU145, the most sensitive to PAC-320 induced cell growth inhibition among the three cell lines, had no detectable Bcl-2. A combination treatment may improve the antitumor effect of PAC-320 to PC3 cells and it needs to further study.

Over the past several years HDACis have been demonstrated to inhibit tumor growth via arrest cell cycle at G2/M phase and/or apoptosis by different mechanism. HDACis SK-7041 and SK-7068 were demonstrated inducing gastric tumor cells G2/M phase arrest, aberrant mitosis and apoptosis because of lacking of G2 CDK inactivation [36]. TSA resulted in a profound reduction in the expression of cyclin A2, cyclin B1/Cdk1 and survivin, thereby induced G2/M cell cycle arrest and apoptosis in Panc1and Capan2 cells [37]. Romidepsin and Vorinostat were demonstrated induce G2 arrest in several human tumor cells [38]. Here, we found that PAC-320 treatment induces cell cycle arrest at G2/M phase. During G2/M, Cdk1 triggers entry into mitosis when bound to cyclin A or cyclin B1, which were considered as key regulatory proteins driving G2 to M phase [39, 40]. Our data showed that PAC-320 treatment significantly reduced the expression of cyclin A and cyclin B1 in LNCaP or DU145 cells. In addition, we found that PAC-320 could increase p21 expression through enhancing histone acetylation thereby activating its promoter. p21 can suppress cyclin B1 and cdc2 expression by inhibiting either cdc2 kinase activity or blocking the interaction of cyclin B1-cdc2 complexes with their substrates, leading to G2/M-phase cell cycle arrest [41, 42]. The role of p21 in apoptosis is controversial. In some tumors, induction of p21 expression also correlates with apoptosis [43–45]. The role of p21 in PAC-320 induced growth inhibition in human prostate cells needs to further investigate.

We also demonstrated that PAC-320 increases BH3-only proteins Noxa and Hrk and activates mitochondria mediated apoptosis. BH3-only proteins are considered to be essential initiators of the mitochondrial apoptotic pathway [46]. These proteins could activate Bax/Bak directly or indirectly, thereby permeabilize the mitochondrial outer membrane and activate intrinsic apoptosis [28]. FK-228 and Vorinostat were reported could induced hyperacetylation of BH3-only protein promoter and enhance Noxa expression [47–49]. Noxa is a p53 target, and they showed that Noxa increases after HDACi treatment is p53-dependent [48]. Here, we showed that Noxa could be induced by PAC-320 in LNCaP or DU145 cells. Because wild type p53 in LNCaP cells and mutated p53 in DU145 cells were decreased after PAC-320 treatment, it should be existed another way to induce Noxa expression. Hrk was also be induced by treatment with HDACis, such as TSA, depsipeptide or KBH-A42 [50, 51]. Mitochondria mediated apoptosis after PAC-320 treatment is activated, at least partially, by upregulation of BH3-only proteins Noxa and Hrk.

p38 MAPK is mainly activated by stress stimuli and mediates signals that regulate various cellular responses, including cell-cycle progression and apoptosis, depending on cell types and stimuli [52]. Several HDACis, such as TSA, MS-275 and FK228, has been reported to activate p38 MAPK pathway in various cell lines [30–32]. We noted that p38 MAPK was activated after PAC-320 treatment. p38 MAPK signaling inhibition partially restored PAC-320 induced decrease in cell viability in DU145 cells. Therefore, except for p38 MAPK, other signaling pathways may be involved in growth inhibition by PAC-320.

Together with our previous study [18], we demonstrated that PAC-320 is a broad-spectrum HDACi at micromole level, and displays attractive antitumor activity against various cancer, but low toxicity to normal cells and animals. PAC-320 is a polyoxometalate compound and exhibits many merits, including simple and easy synthesis procedure, high yield, good water solubility and stability under the physiological conditions. These advantages allow for further investigation of the biological activity of PAC-320 for potential cancer chemotherapeutics.

In conclusion, we demonstrated that POM-based HDACi PAC-320 could inhibit growth of human prostate cancer cells *in vitro* and *in vivo*. Furthermore, PAC-320 induces G2/M arrest and mitochondria mediated apoptosis in prostate cancer cells. These events are accompanied by induction of several pro-apoptosis and cell cycle-related proteins, Noxa, Hrk and p21, and reduction of G2/M transition associated proteins, cyclin A and cyclin B1. The present findings raise the possibility that PAC-320 may prove particularly effective in the treatment of prostate cancers.

## MATERIALS AND METHODS

### Cells and reagents

Human prostate cancer cell lines LNCaP, DU145 and PC3 were obtained from Shanghai Cell Line Bank, Chinese Academy of Sciences. Cells were grown in RPMI 1640 medium supplemented with 10% fetal bovine serum and were incubated under standard culture condition (5% CO_2_; 37°C). SAHA was purchased from Selleck, and sodium butyrate (NaB) was purchased from Bio Basic Inc. (BBI). p38 inhibitor SB203580 was purchase from Calbiochem.

### Antibodies

Rabbit α-p21 (#sc-397), rabbit α-cyclin A (#sc-596) and rabbit α-cyclin B1 (#sc-4073) were purchased from Santa Cruz Biotechnology. Rabbit α-acetyl H3 (#06-599) was purchased from Merck Millipore. Mouse anti-β-actin (#A1978) was purchased from Sigma Aldrich. Rabbit α-p38 (#9212), Rabbit α-phospho-p38 (#9211), Rabbit α-ERK1/2 (#9102), Rabbit α-phospho-ERK1/2 (#9101), Rabbit α-JNK (#9252), Rabbit α-phospho-JNK (#9251), Rabbit α-cleaved caspase 3 (#9664), Rabbit α-cleaved caspase 7 (#8438), and Rabbit α-cytochrome c (#4272) were purchase from Cell Signaling Technology. Rabbit α-Hrk (#GTX54049) was purchase from GeneTex. Rabbit α-Noxa (#ab36833) was purchase from Abcam. Rabbit α-PARP1 (#13371-1-AP) was purchased from ProteinTech.

### HDAC activity assay

The HDAC inhibition assay was performed using an HDAC fluorescent activity assay kit (BIOMOL, Inc.), following the manufacturer’s recommendations. In brief, synthetic HDAC substrates were added to purify HDACs in the presence or absence of PAC-320, and the developer was added after a 30 mins reaction. Deacetylation of the substrate sensitizes it to the developer, which then generates a fluorophore. The fluorophore was excited with 360 nm light and the emitted light (460 nm) was detected on an LS55 luminescence spectrometer (PerkineElmer).

### Cell proliferation (MTT) assay, cell cycle analysis and apoptosis assay

For cell proliferation assay, 3000 cells were seeded on 96-well plates and incubated for 24 h and treated with drugs at indicated concentrations for 3 days at 37°C. After drug treatment, 3-(4,5-dimethylthiazol-2-yl)-2,5-diphenyltetrazolium bromide (MTT) solution was added to each well and incubated for 4 h at 37°C before media removal. DMSO was then added and shaken for 30 min at room temperature. Cell viability was determined by measuring the absorbance at 492 nm. Values represent the mean of 3 experiments (bars, ± SD).

For cell cycle analysis, cells were treated with PAC-320 for 48 h and fixed in 70% ethanol/30% PBS for 1 h at –20°C. After fixation, cells were washed once with PBS, resuspended, and incubated in propidium iodide (PI) buffer (60 μg/ml PI and 0.1 mg/ml RNase A) for 45 min at room temperature. Fluorescent-activated cell sorting (FACS) was conducted on at least 5,000 cells per condition using a BD FACSVerse system and BD FACSuite software. Cell cycle profiles were processed and analyzed using FlowJo version 6.4.7 (Tree Star, Ashland, OR, USA).

For apoptosis assay, cells were treated with PAC-320 for 48 h and harvested. Cells were stained as described in the Annexin V FITC Apoptosis Detection Kit (KeyGen Biotech) and data was acquired using a BD FACSVerse system and BD FACSuite software.

### Subcellular fractionation

Cells were treated with or without PAC-320 and cytosolic proteins were fractionated as described previously [53]. Briefly, cells were resuspended in a lysis buffer containing 0.025% digitonin, sucrose (250 mM), HEPES (20 mM; pH 7.4), MgCl_2_ (5 mM), KCl (10 mM), EDTA (1 mM), phenylmethylsulfonyl fluoride (1 mM), 10 μg/mL aprotinin, 10 μg/mL leupeptin. After 10 min incubation at 4°C, cells were centrifuged (2 min at 13,000 × g) and the supernatant (cytosolic fraction) was removed and frozen at -80°C for subsequent use.

### Immunoblot analysis

Cells were harvested after treatments and lysed in the lysis buffer for 30 min at 4°C. Total cell extracts were separated in SDS- polyacrylamide gel electrophoresis (PAGE), and then transferred to a polyvinylidene fluoride membrane. The membrane was incubated with indicated antibodies. The signals were visualized by using the Chemiluminescent Substrate method with the SuperSignal West Pico kit provided by Pierce Co and densitometries of electrophoretic bands were quantitated by using Scion Image software.

### RT-PCR and real-time PCR

Total RNA was isolated using the Trizol reagent (Invitrogen) following manufacturer’s instructions. One microgram RNA was used for cDNA synthesis using a reverse transcriptase reaction kit (Promega). RT-PCR and Quantitative real-time PCR was performed on an ABI Prism 7000 Sequence Detection System (Applied Biosystems), using SYBR Green (TIANGEN BIOTECH) as a dsDNA-specific fluorescent dye. The PCR primer sequences were as follows. p21: 5′-GGATGTCCGTCAGAACCC-3′ (sense) and 5′-GCTCCCAGGCGAAGTCA-3′ (antisense); Hrk: 5′-CAGGCGGAACTTGTAGGAAC-3′ (sense) and 5′-GCTGGATTTCCAAAGGGCTT-3′ (antisense); Noxa: 5′-GTGCCCTTGGAAACGGAAGA-3′ (sense) and 5′-CCAGCCGCCCAGTCTAATCA-3′ (antisense); Bcl-xl: 5′-TCCCAGAAAGGATACAGCTGG-3′ (sense) and 5′-ACTGAAGAGTGAGCCCAGCAG-3′ (antisense); Bik: 5′-TCATGGACGGTTTCACCACA-3′ (sense) and 5′-CAGTGTTCCAGCACTATCTC-3′ (antisense); Bid: 5′-ACCCTAGAGACATGGAGAAG-3′ (sense) and 5′-AGCTATCTTCCAGCCTGTCT-3′ (antisense); and β-actin: 5′-TCGTGCGTGACATTAAGGAG-3′ (sense) and 5′-ATGCCAGGGTACATGGTGGT-3′ (antisense). Data were analyzed by using the 2^–ΔΔCt^ method [54]. RT-PCR products were separated by agarose gel electrophoresis and densitometries of electrophoretic bands were quantitated by using Scion Image software. All results represent means ± standard deviations of three independent experiments.

### Chromatin immunoprecipitation (ChIP) assay

ChIP analysis was performed using the ChIP Assay Kit (upstate) according to manufacturer’s protocol. Immunoprecipitation was done with rabbit α-acetyl H3 antibody. Primer pairs for *p21* proximal promoter were: P1: 5′-GGTGTCTAGGTGCTCCAGGT-3′ (sense) and 5′- GCACTCTCCAGGAGGACACA-3′ (antisense); P2: 5′- CGTGGTGGTGGTGAGCTA -3′ (sense) and 5′- CTGTCTGCACCTTCGCTCCT -3′ (antisense); for p21 distal promoter were 5′-AATTCCTCTGAAAGCTGACTGCC-3′ (sense) and 5′- AGGTTTACCTGGGGTCTTTAGA-3′ (antisense). PCR products were separated by agarose gel electrophoresis.

### Assay of caspase 3/7 activity

LNCaP or DU145 cells were plated at 2 × 10^3^ cells per well on 96-well plates. At 24 h after treatment with PAC-320, the Apo-ONE caspase 3/7 Reagent (Promega, Madison/Wisconsin) was added. Cells were incubated for 1 h at room temperature prior to record of the fluorescence (485Ex/527Em).

### Determination of mitochondrial membrane potential

Mitochondrial membrane potential (MMP) was measured using a lipophilic cationic fluorochrome, JC-1 (BD Biosciences, San Jose, CA). It forms aggregates (red fluorescent, FL2) at high dye concentrations at normal MMP and forms monomers (green fluorescent, FL1) at lower concentrations as MMP is lost. Therefore, depolarization of MMP is indicated by a decrease in the ratio of red/green fluorescence intensity. CCCP (carbonyl cyanide 3-chlorophenylhydrazone), a MMP disrupter, was used as positive control. Cells were incubated with JC-1 staining solution for 15 min at 37°C, then rinsed twice with assay buffer and analyzed by FACS.

### *In vivo* antitumor activity

The human prostate cancer cell line DU145 were xenografted s.c. into the right subaxillary region of nude mice and maintained by the serial s.c. transplantation of 3-mm^3^ fragments. Mice bearing a tumor xenograft were randomized into treated and control groups of 6 mice per group. Treatment was initiated at ∼2 weeks after transplantation, when tumors reached a weight of 40–60 mm^3^. Compounds (PAC-320, NaB or SAHA) were administered i.p. per day for 16 days at an indicated dose. Tumors were measured using a Vernier caliper, and tumor volume (V) was calculated daily using the equation V = 1/2ab^2^, where a and b represent the length and width, respectively (in millimeters). On day 17 after treatment, the mean tumor weight, relative tumor growth, and inhibition rate were determined. Relative tumor growth was calculated relative to the initial starting volume for each individual mouse. Inhibition rate was obtained from the equation (1-relative tumor growth in treated/relative tumor growth in control) × 100.

### Statistical analysis

SPSS 21.0 was used for statistical analysis. The significance level was set as *P, ^#^*P* < 0.05; **P, ^*##*^*P* < 0.01; ***P, ^*##*^*P* < 0.001.

## SUPPLEMENTARY MATERIALS FIGURES


