# Supplementary material for: HDAC inhibitor PAC-320 induces G2/M cell cycle arrest and apoptosis in human prostate cancer
Source: Oncotarget. 2017 Dec 8;9(1):512–23. doi: 10.18632/oncotarget.23070 (PMC5787485; doi:10.18632/oncotarget.23070)
Supplement: Supplementary file 1 [file oncotarget-09-512-s001.pdf]

HDAC inhibitor PAC-320 induces G2/M cell cycle arrest and apoptosis in human prostate cancer

SUPPLEMENTARY MATERIALS

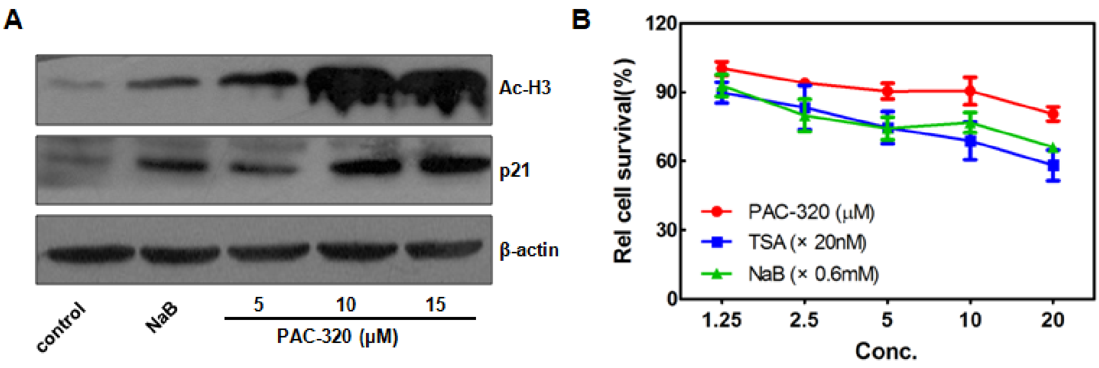

Supplementary Figure 1: The effects of PAC-320 on PC3 cells. (**A**) PAC-320 increases the level of acetylated H3 in PC3 cells. Immunoblotting analyze the effect of PAC-320 on acetylation of histone H3 in PC3 cells. NaB as a positive control. (**B**) Antiproliferative effect of PAC-320 on PC3 cells. PC3 cells were treated with indicated drugs for 72 h, and the effects of PAC-320 on the proliferation of PC3 cells were determined using the MTT assay. TSA and NaB were used as positive controls. Each value represents the mean of three experiments; bars, ± SD.

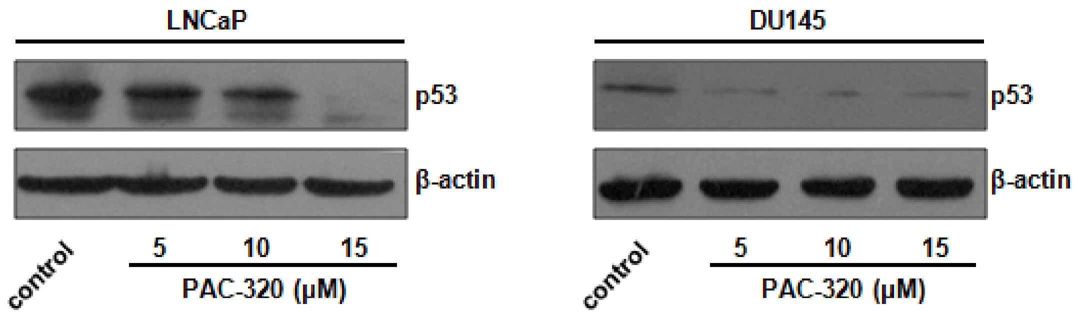

Supplementary Figure 2: PAC-320 decreases p53 expression in prostate cancer cells. LNCaP (**A**) or DU145 (**B**) cells were treated with indicated concentrations of PAC-320 for 48 h, and whole cell lysates were immunoblotted with indicated antibodies.

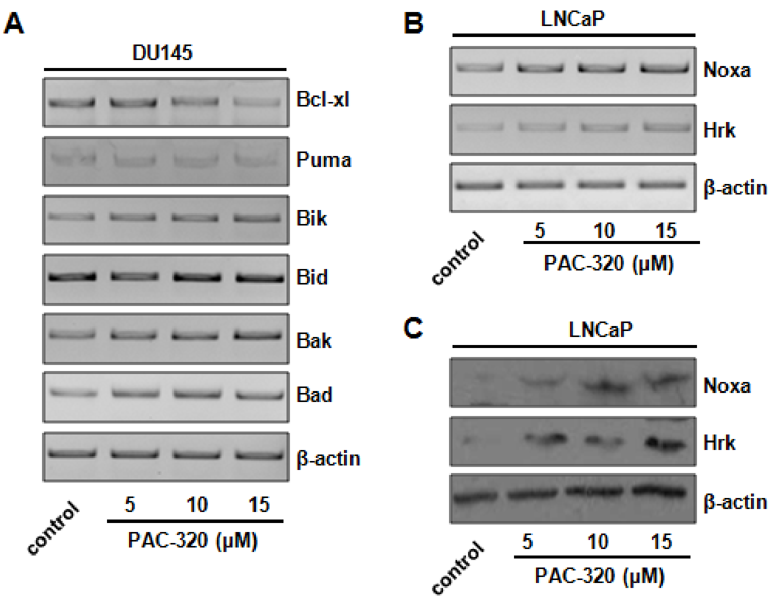

Supplementary Figure 3: The effects of PAC-320 on BH3-only proteins in prostate cancer cells. (**A**) The effects of PAC-320 on expression of BH3-only proteins in DU145 cells. DU145 cells were treated with indicated concentrations of PAC-320 for 48 h. The mRNA levels of BH3-only proteins were analyzed using RT-PCR. (**B**–**C**) The effects of PAC-320 on expression of Noxa and Hrk in LNCaP cells. LNCaP cells were treated with indicated concentrations of PAC-320 for 48 h. The expression of Noxa and Hrk were analyzed using RT-PCR (B) or Immunoblot (C).

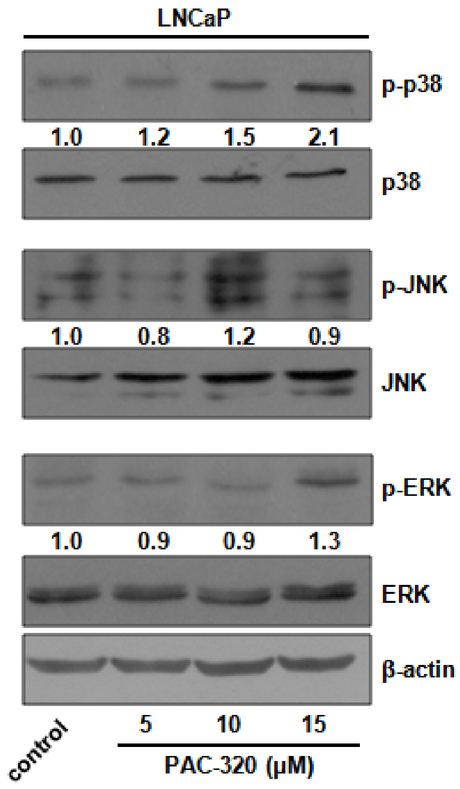

Supplementary Figure 4: The effects of PAC-320 on MAPK activation in LNCaP cells. LNCaP cells were treated with indicated concentrations of PAC-320 for 48 h, and whole cell lysates were immunoblotted with indicated antibodies. Numbers below the panels of the immunoblot indicated the protein levels relative to β-actin.
